# Long-term psychosocial impact of alternative management policies in women with low-grade abnormal cervical cytology referred for colposcopy: a randomised controlled trial

**DOI:** 10.1038/sj.bjc.6606042

**Published:** 2010-12-21

**Authors:** L Sharp, S Cotton, N Gray, M Avis, I Russell, L Walker, N Waugh, D Whynes, C Woolley, A Thornton, L Smart, M Cruickshank, J Little

**Affiliations:** 1 National Cancer Registry, Building 6800, Cork Airport Business Park, Kinsale Road, Cork, Ireland; 2 Department of Public Health, University of Aberdeen, Aberdeen, Scotland; 3 Centre for Academic Primary Care, University of Aberdeen, Aberdeen, Scotland; 4 Department of Nursing Studies, University of Nottingham, Nottingham, England; 5 Department of Primary Care and Public Health, University of Bangor, Bangor, Wales; 6 Institute of Rehabilitation, University of Hull, Hull, England; 7 School of Economics, University of Nottingham, Nottingham, England; 8 Department of Pathology, University of Aberdeen, Aberdeen, Scotland; 9 Department of Obstetrics & Gynaecology, University of Aberdeen, Aberdeen, Scotland; 10 Department of Epidemiology and Community Medicine, University of Ottawa, Ottawa, Canada

**Keywords:** anxiety, colposcopy, depression, distress, LLETZ, punch biopsies

## Abstract

**Background:**

The debate continues regarding the best management for women with low-grade abnormal cervical cytology attending colposcopy. We compared psychosocial outcomes of alternative management policies in these women.

**Methods:**

In all, 989 women, aged 20–59 years, with low-grade abnormal cytology, were randomised to immediate large loop excision (LLETZ) or two to four targeted punch biopsies taken immediately with recall for LLETZ if these showed cervical intra-epithelial neoplasia 2/3. At 6 weeks after the last procedure, women completed the hospital anxiety and depression scale (HADS) and the impact of event scale (IES). At 12, 18, 24 and 30 months post recruitment, women completed the HADS and process outcome specific measure (POSM). Prevalence of significant depression (⩾8), significant anxiety (⩾11) and distress (⩾9) and median POSM scores were compared between arms. Multivariate odds ratios (ORs) for immediate LLETZ *vs* biopsy and recall were computed.

**Results:**

Over the entire follow-up, there was no significant difference between arms in cumulative prevalence or risk of significant depression (OR=0.78, 95% CI 0.52–1.17) or significant anxiety (OR=0.83, 95% CI 0.57–1.19). At 6 weeks post procedure, distress did not differ significantly between arms. At later time points, 8–11% had significant depression and 14–16% had significant anxiety but with no differences between arms. The POSM scores did not differ between the arms.

**Conclusions:**

There is no difference in long- or short-term psychosocial outcomes of immediate LLETZ and punch biopsies with selective recall.

The receipt of a low-grade abnormal cervical cytology test result, and subsequent follow-up investigations and treatment, can have adverse psychosocial consequences for women (Bell *et al*, 1995; Maissi *et al*, 2004; Gray *et al*, 2006). For instance, women with low-grade abnormal cytology are increasingly referred for a colposcopy examination (TOMBOLA Group, 2006), an event which is associated with considerable procedural distress (Posner and Vessey, 1988). Several studies have shown that women have raised anxiety levels before and during colposcopy (see e.g., Galaal *et al*, 2007; Hellsten *et al*, 2007; Tahseen and Reid, 2008). Less is known about psychosocial outcomes among women in the months and years after colposcopy.

The most effective management of women with low-grade abnormal cytology who have a visible abnormality at colposcopy is controversial. Although immediate treatment by large loop excision (LLETZ; ‘see-and-treat’) removes all grades of cervical intra-epithelial neoplasia (CIN), enables full histological examination, and minimises the possibility of default from follow-up, it can result in overtreatment (Holschneider *et al*, 1999; Cardenas-Turanzas *et al*, 2005; van Hamont *et al*, 2006). In addition, LLETZ has been associated with subsequent raised risk of pre-term delivery (Noehr *et al*, 2009). On the other hand, targeted punch biopsies with selective recall for treatment of those with histologically confirmed high-grade disease may miss prevalent CIN and place women with untreated CIN1 at risk of subsequently developing high-grade lesions (Cox *et al*, 2003; Byrom *et al*, 2006). So far, there has been little consideration of the psychosocial sequelae of these management strategies. The single available observational study found that, at 7 days post colposcopy, women with a colposcopic impression of high-grade disease who had been managed by immediate LLETZ reported lower anxiety and higher relief and perception of control than those managed by biopsy and selective recall (Orbell *et al*, 2004; Balasubramani *et al*, 2007).

The United Kingdom TOMBOLA trial was the first randomised controlled trial to evaluate immediate LLETZ *vs* targeted punch biopsies with selected recall in women with low-grade cervical cytology (Cotton *et al*, 2006). We found that detection rates of CIN2 or more severe disease over 3 years did not differ significantly between the policies (TOMBOLA Group, 2009a). In this study, we compare the psychosocial impact on women over a 30-month period following the management interventions.

## Subjects and Methods

### Subjects and recruitment

Subject eligibility and recruitment processes are described in detail elsewhere (Cotton *et al*, 2006; TOMBOLA Group, 2009b). Briefly, all participants had been called for a routine cytology test as part of the NHS Cervical Screening Programmes, and attended for that test during October 1999 to October 2002. Eligible women were aged 20–59 years, had low-grade changes (mild dyskaryosis or borderline nuclear abnormalities (BNA)), were not pregnant and had no previous cervical treatment. Women were invited to hospital-based recruitment clinics where they were recruited by non-clinical staff. Consenting women provided a sample which was tested for high-risk HPV types; neither the specific purpose of the sample nor the test result was disclosed to women or clinicians involved in their management. Women were subsequently randomised to cytological surveillance or initial colposcopy using a telephone service provided by Aberdeen University. Those allocated to colposcopy were sent an appointment to attend a hospital-based colposcopy clinic.

### Procedures and follow-up

During the colposcopy appointment, but before the colposcopic examination itself, consenting women were randomised to biopsy and selective recall or immediate LLETZ. Randomisation was stratified by centre, age group, cytology grade and high-risk HPV status. When the examination was undertaken, the colposcopist was aware of the arm to which the woman was allocated. Of women with adequate colposcopy, those with an abnormal transformation zone received the intervention assigned by randomisation, whereas those whose transformation zone was normal were followed-up by 12-monthly cervical cytology tests in primary care. Women with inadequate colposcopy were excluded from the comparison of the management policies and were treated according to local NHS protocols.

For biopsy and selective recall, up to four targeted punch biopsies were taken from the most abnormal areas. Women with CIN2/3 on histology were recalled for treatment by LLETZ. Women with no CIN or CIN1 on histology did not receive any further treatment at this time and were followed-up in primary care by 6-monthly cytology tests. In the other arm, the whole transformation zone, including the abnormality, was removed immediately by LLETZ. Follow-up after punch biopsies or loop excision was by cytology tests in primary care every 6 months (Cotton *et al*, 2006). Cytology follow-up results were monitored with subsequent action (next recommended test date or colposcopy referral) based on these results. If women were referred for colposcopy during the follow-up, they attended local NHS colposcopy clinics and were treated, if required, according to the local protocols. Approximately 3 years following recruitment, women were invited for an exit colposcopy examination.

### Psychosocial assessments

**Figure 1:**
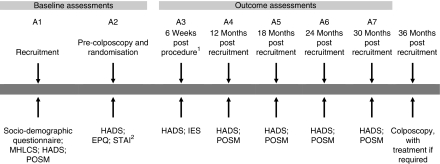
Timing of events and psychosocial assessments, and instruments included. EPQ, Eysenck personality questionnaire; HADS, hospital anxiety and depression scale; IES, impact of event scale; MHLCS, multi-dimensional health locus of control scale; POSM, process outcome specific measure, STAI, Spielberger state–trait anxiety inventory. ^1^At 6 weeks after colposcopy, punch biopsy(ies) or LLETZ, whichever took place last. For women with a normal transformation zone at colposcopy, those who had immediate loop excision, and those who had punch biopsies which showed CIN0/1, this was 6 weeks after the initial (and only) colposcopy; for those women who had punch biopsies which showed CIN2/3 and were recalled for LLETZ, this was 6 weeks after the treatment appointment. ^2^All administered at colposcopy appointment, before examination and randomisation.

Women recruited to TOMBOLA from February 2001 onwards, who underwent colposcopy during or after December 2001, and who consented to the second randomisation (*n*=989) were asked to complete seven psychosocial assessments (A1–A7; Figure 1). These assessments required the completion of psychosocial questionnaires at recruitment (A1), during the colposcopy appointment (before the examination and the second randomisation (A2)), 6 weeks after colposcopy and related interventions (A3), and at 12 (A4), 18 (A5), 24 (A6) and 30 months (A7) after recruitment. The recruitment and pre-colposcopy assessments (A1 and A2) provided data on potential explanatory variables/confounders. Outcome information was obtained from the assessments undertaken after the second randomisation (A3–A7). The 6-week assessment was designed to evaluate short-term psychosocial effects after the completion of treatment. The timing of the assessments of long-term effects (12, 18, 24 and 30 months) was designed not to be close to the expected follow-up visits (cytology tests or colposcopy), thus avoiding detecting ‘spikes’ of anxiety associated with these.

The main outcomes were depression and anxiety over the long-term as assessed by the hospital anxiety and depression scale (HADS; Zigmond and Snaith, 1983), with secondary outcomes assessed using the process outcome specific measure (POSM; Gray *et al*, 2005) and the impact of event scale (IES; Horowitz *et al*, 1979). The HADS was originally designed to screen for clinically significant anxiety and depression in hospital outpatient clinics, but has subsequently been validated in primary care and community settings (Snaith, 2003). It discriminates well between groups with different prevalence of anxiety and depressive disorders and is responsive to temporal changes (Herrmann, 1997; Bjelland *et al*, 2002). Women completed the HADS at all seven time points. As the HADS may fail to detect subtle, but important, psychosocial consequences of receiving an abnormal cytology test and its subsequent management, women were also asked to complete the POSM at the A4–A7 time points. This instrument was specially developed for use in TOMBOLA. It includes 16 questions addressing a range of issues, including cancer, health, fertility and sexual concerns, and has acceptable psychometric properties (Gray *et al*, 2005). The IES measures stress reactions after a specific traumatic event, and has been validated and used in a variety of contexts (Sundin and Horowitz, 2002, 2003). It assesses overall distress and, in subscales, intrusive experiences and avoidance of thoughts or images associated with the event. The IES was included in the 6-week questionnaire (A3).

The recruitment questionnaire (A1) included a section on socio-demographic and lifestyle factors (such as, educational level, employment status, parity and smoking) and the multi-dimensional health locus of control scale (MHLCS), which measures three dimensions of health locus of control (chance, internal and powerful others; Wallston *et al*, 1978). The pre-colposcopy questionnaire (A2) included the short form of the Spielberger state–trait anxiety inventory, which measures anticipatory anxiety (Marteau and Bekker, 1992), and Eysenck's short questionnaire, which measures two dimensions of personality (extraversion–introversion, neuroticism–stability; EPQ; Eysenck, 1958).

### Statistical analysis

All analyses were by intention-to-treat. Questionnaire response rates were based on the total number of women randomised in each arm and are thus conservative. The HADS, POSM and IES scores were not normally distributed. For HADS and IES, therefore, individuals were defined as ‘cases’ or ‘non-cases’, depending on whether they scored above or below specific values on each subscale/instrument. In the primary analysis, ‘significant depression’ was defined as a HADS depression subscale score ⩾8. This cut-off has been recommended in guidelines for detecting depression in cancer patients (Rodin *et al*, 2006) and used in previous research among women with abnormal cervical cytology (Bell *et al*, 1995). Following Zigmond and Snaith (1983), ‘significant anxiety’ was defined as a HADS anxiety subscale score ⩾11. Women with a total IES score of ⩾9 were classified as distressed, with subcategories of mild (total score 9–25), moderate (26–43) and severe (⩾44). Following common practise (Salvesen *et al*, 1997; Broen *et al*, 2005), women scoring ⩾20 on the avoidance or intrusion subscales of the IES were defined as cases. The total POSM score was computed for each woman (Gray *et al*, 2005) and categorised at the median value for analysis (low<median, high⩾median).

The point prevalence of significant depression, significant anxiety, distress, avoidance and intrusion was calculated at all seven time points for (1) all women (i.e., combining trial arms) and (2) by arm. The analysis combining trial arms was carried out to assess the temporal trends in significant depression and anxiety. The point prevalence of significant depression and significant anxiety in all women was compared between consecutive time points using *z*-tests. The analysis by arm compared the effects of the management policies. Using data from the A3–A7 time points, the cumulative prevalence of significant depression and anxiety in each arm was computed (i.e., the percentage of women who scored in the range for significant depression or significant anxiety at one or more time point). Cumulative and point prevalence (i.e., at each individual time point) was compared between arms using *z*-tests. The Mann–Whitney/Wilcoxon test was used to compare total POSM scores in all women from one time point to the next and between arms at each time point. Logistic regression methods were used to compute odds ratios (ORs) for immediate LLETZ *vs* biopsy and selective recall. Separate models were built for each outcome. Risk estimates were adjusted for the randomisation stratification variables (adjusted ORs), and then for significant confounders from among the socio-demographic, lifestyle and psychosocial variables collected at recruitment and pre-colposcopy (multivariate ORs; candidate confounders are shown in Table 1). Confounders were included in the multivariate models if they were significant (*P*<0.1) on likelihood ratio tests. Final models had adequate fit as assessed by the test of Hosmer and Lemeshow (1989).

The analyses included only those women who completed all questions on the relevant sub-scale/instrument. The number of women excluded at each time point because of the missing items was small (HADS: <5 in each arm; IES: <20 in each arm; POSM <40 in each arm).

Sensitivity analyses were conducted to explore the impact on the results of the cut-offs used to define ‘caseness’. The analyses described above were repeated using alternative cut-offs. These were: significant depression ⩾11; significant anxiety ⩾8; and distress ⩾25 and ⩾44. To explore whether there was evidence of participation bias, the primary analyses were repeated restricting the study population to (1) women who had completed the psychosocial questionnaire at every point from A3 to A7, and (2) women who had completed the psychosocial questionnaire at only one time point during A3 to A7.

### Ethical approval

Ethical approval was obtained from the joint Research Ethics Committee of NHS Grampian and the University of Aberdeen, the Tayside Committee on Medical Research Ethics and the Nottingham Research Ethics Committee. This approval required that we inform the GP of any woman who scored ⩾8 on the HADS depression subscale at any time point.

## Results

### Baseline characteristics of participants

**Figure 2:**
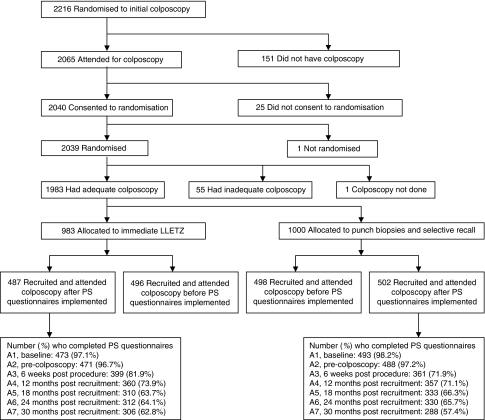
Numbers of women randomised and included in the psychosocial (PS) comparison.

**Figure 3:**
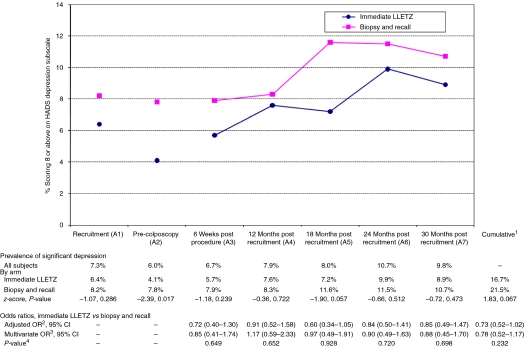
Prevalence of significant depression (HADS depression sub-scale score of ⩾8), with odds ratios (ORs), 95% confidence intervals (CIs), and *P*-values, by randomisation arm. ^1^Significant depression at any of the outcome assessment points, A3–A7. ^2^Adjusted for randomisation minimisation variables (age group, trial centre, high-risk HPV status and recruitment cytology status). ^3^Adjusted for minimisation variables, and the following: 6 weeks, ever had children, HADS depression pre-colposcopy, HADS anxiety pre-colposcopy, EPQ neuroticism, MHLCS chance; 12-months, ever had children, HADS depression pre-colposcopy, HADS anxiety pre-colposcopy, EPQ neuroticism; 18 months, HADS depression pre-colposcopy, EPQ neuroticism smoking status; 24-months, ever had children, HADS depression pre-colposcopy, HADS anxiety pre-colposcopy, EPQ neuroticism; 30 months, HADS depression pre-colposcopy, HADS anxiety pre-colposcopy, smoking status; cumulative, HADS anxiety pre-colposcopy, HADS depression pre-colposcopy, EPQ neuroticism, smoking status, ever had children, ethnic group, employment status. ^4^Likelihood ratio test *P*-value for randomisation arm, from multivariate model.

**Figure 4:**
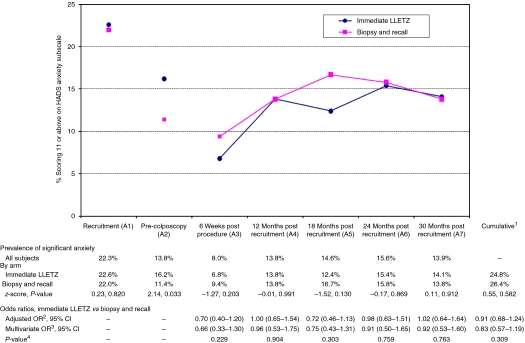
Prevalence of significant anxiety (HADS depression sub-scale score of ⩾11), with odds ratios (ORs), 95% confidence intervals (CIs), and *P*-values, by randomisation arm. ^1^Significant anxiety at any of the outcome assessment points, A3–A7. ^2^Adjusted for randomisation minimisation variables (age group, trial centre, high-risk HPV status and recruitment cytology status). ^3^Adjusted for minimisation variables, and the following: 6 weeks, ever had children, HADS anxiety pre-colposcopy, EPQ neuroticism, MHLCS chance; 12-months, ever had children, deprivation category, HADS depression pre-colposcopy, HADS anxiety pre-colposcopy, STAI pre-colposcopy, EPQ neuroticism; 18 months, HADS anxiety pre-colposcopy, HADS depression pre-colposcopy, STAI pre-colposcopy, EPQ neuroticism; 24-months, smoking status, HADS depression pre-colposcopy, HADS anxiety pre-colposcopy, STAI pre-colposcopy, EPQ neuroticism; 30 months, ever had children, HADS anxiety pre-colposcopy, EPQ neuroticism, MHLCS chance; cumulative, HADS anxiety pre-colposcopy, HADS depression pre-colposcopy, EPQ neuroticism, ever had children, employment status. ^4^Likelihood ratio test *P*-value for randomisation arm, from multivariate model.

**Figure 5:**
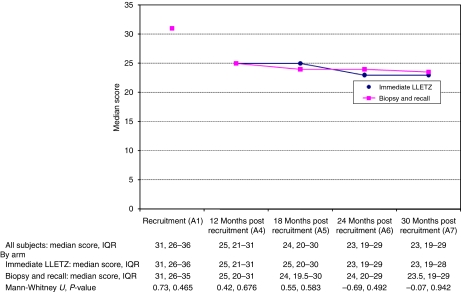
The POSM median scores, with *P*-values, by randomisation arm. IQR, interquartile range.

In all, 989 women were eligible to take part, 487 of whom were randomised to immediate LLETZ and 502 to biopsy and selective recall (Figure 2). In all, 43% were aged 20–29, 27% aged 30–39, 21% aged 40–49 and 9% aged 50–59. Around one-quarter had mild dyskaryosis at recruitment. The majority of women were white and in full or part-time employment, and just over one-quarter had obtained a college/university degree. Slightly more than one-third were current smokers. One-third had never been pregnant. The arms were balanced in terms of the randomisation stratification variables (age group, recruitment cytology, centre and high-risk HPV status), the socio-demographic and lifestyle factors and psychosocial characteristics (MHLCS, EPQ) assessed at recruitment (A1; Table 1), and prevalence of significant depression (HADS subscale score ⩾8) and significant anxiety (HADS subscale score ⩾11) and median POSM scores at recruitment (Figures 3, 4 and 5). At the pre-colposcopy psychosocial assessment (A2), significant depression was more common in the biopsy and recall arm (7.8 *vs* 4.1%), significant anxiety was more common in the immediate LLETZ arm (16.2 *vs* 11.4%), and the median POSM scores were identical (Figures 3, 4 and 5).

### Response rates

At recruitment and pre-colposcopy at least 97% of women in each arm completed the psychosocial questionnaire (Figure 1). At 6 weeks post procedure (A3), the response rate was higher in the immediate LLETZ arm (82 *vs* 72% *z*=3.73, *P*<0.01). Response rates fell at subsequent time points (to between 57 and 74%), but did not differ significantly between the arms at any point.

### Hospital anxiety and depression scale: depression

Considering all women, the point prevalence of significant depression was between 6.0 and 10.7% at each assessment point from A1 to A7 (Figure 3), with no time point significantly different from the successive one. On comparing management arms, cumulative prevalence of significant depression over the entire follow-up period was slightly lower in the immediate LLETZ arm (16.7 *vs* 21.5% Figure 3), and this was borderline statistically significant (*P*=0.067), but after adjustment for confounders risk of depression did not differ significantly between arms (multivariate OR=0.78, 95% CI 0.52–1.17). There were no differences between the arms in point prevalence or risk of depression at 6 weeks post procedure (A3) or at any subsequent time point (A4–A7).

### Hospital anxiety and depression scale: anxiety

Considering all women, point prevalence of significant anxiety fell significantly from recruitment (A1: 22.3%) to pre-colposcopy (A2: 13.8% *P*<0.001; Figure 4) and from pre-colposcopy to 6 weeks post procedure (A3: 8.0% *P*<0.001), then returned to 13.8% at 12 months (A4: *P*<0.001) and remained stable thereafter. Cumulative prevalence over the entire follow-up period did not differ between arms (immediate LLETZ=24.8%, biopsy and recall=26.4% multivariate OR=0.83, 95% CI 0.57–1.19; Figure 4). There were no significant differences in point prevalence or risk of anxiety between the arms at any of the individual time points from A3 to A7.

### Process outcome specific measure

In the entire group, the median POSM score fell significantly from recruitment to 12 months (28 at A1 *vs* 25 at A4; *P*<0.001) and between 12 and 18 months (24 at A5; *P*<0.001), and remained stable thereafter. It did not differ significantly between arms at any time point from 12 to 30 months (A4–A7; Figure 5).

### Impact of event scale

There was no difference between arms in the point prevalence of distress at 6 weeks post procedure (31.3 *vs* 31.5% Table 2), or in the distribution of distress scores. In the immediate LLETZ arm, 23.1% had mild distress (IES score 9–25); 5.0% had moderate distress (26–43) and 3.2% severe distress (⩾44), compared with 22.8, 6.9 and 1.8% in the biopsy and recall arm (*χ*^2^(3df)=2.38, *P*=0.498). There were no significant differences between arms in the percentage, and risk of, scoring ⩾20 on the avoidance or intrusion subscales (Table 2).

### Sensitivity analyses

Using different cut-offs to define caseness had no impact on the overall findings (data not shown). When the analysis was repeated by including only those women who had completed the (1) HADS or (2) POSM at every outcome time point, the results were unchanged (data not shown). Restricting the analysis to women who had completed the (1) HADS or (2) POSM at a single outcome time point did not affect the results (data not shown).

## Discussion

### Strengths and limitations

The major strengths of this study include the randomised design and the large size. In addition, the TOMBOLA trial was nested within the United Kingdom Cervical Screening Programmes that are population-based and free at the point of delivery. The management policies evaluated were structured to mimic how they would be delivered in clinical practice, and other than the recruitment HPV test, no non-routine interventions were made during the psychosocial follow-up. In all, 52% of eligible women participated in TOMBOLA, which compares favourably with population-based epidemiological studies (Olson, 2001), especially in view of concerns about barriers to participation of women in trials (Sharp *et al*, 2006). Participation was lower among younger than older women (TOMBOLA Group, 2009b). As we have shown that, among women with low-grade abnormal cervical cytology, the prevalence of anxiety decreases with increasing age (Gray *et al*, 2006), our results may underestimate the overall frequency of significant anxiety in this population. However, the internal comparison of the management arms is entirely valid. In addition, the ratio of BNA to mild cytology results at trial recruitment was close to United Kingdom population figures (TOMBOLA Group, 2009a), and the incidence of CIN2 or worse over 3 years was similar to other studies nested within the United Kingdom screening programmes (Rana *et al*, 2004; Smith *et al*, 2006). This suggests that generalisibility of the findings is likely to be high.

We assessed a range of psychosocial outcomes using well-established and validated instruments (HADS and IES) with good psychometric properties. The POSM was developed through an extensive process, including literature review and focus groups, and captures different sequelae than the HADS (Gray *et al*, 2005). Unlike most of the previous studies, we also assessed the long-term psychosocial outcomes of management, and carefully timed assessments to avoid being unduly influenced by short-term affects of attending for follow-up tests or receipt of results.

It is possible that women's knowledge and understanding of abnormal cervical cytology results and cervical cancer at recruitment might have influenced the psychosocial impact of the management options following colposcopy, but randomisation should have balanced these factors between the arms. As the trial was pragmatic in design, we did not standardise the information that women were given by the health professionals involved in their care (other than the information leaflets provided at recruitment and before the randomisation). It is likely that there was considerable heterogeneity in the amount and content of the information women sought and obtained during trial participation, both from health professionals and other sources (e.g., the internet), and their understanding of this information. However, all of the trial colposcopists treated women in both arms, and it would be expected that the health- and information-seeking behaviour of women in the two arms would be similar. Therefore, we do not expect these factors to have strongly affected the comparison of the management policies.

Questionnaire response rates were highest at the 6-week assessment (A3) and decreased over time. Our analysis of individual time points included women who responded at that time point. Other than at the 6-week assessment, response rates did not differ by arm at any time point. The socio-demographic characteristics of responders at each point were similar to those of the entire study population. We examined the possibility that non-responders were women who scored high (or low) on a particular sub-scale/instrument. Slightly lower proportions of women who scored in the range for clinically significant depression or anxiety at recruitment completed all five of the outcome questionnaires (percentage completing all questionnaires for women with depression scores ⩾8, 38% compared with 43% for women with lower depression scores; for anxiety ⩾11, 38% compared with 44%,), but these differences were not statistically significant. Nor was there any significant association between the number of outcome questionnaires completed (i.e., 0–5) by depression or anxiety level at recruitment. Moreover, there were no significant differences in these associations between the trial arms. In addition, our sensitivity analysis showed that the results were unchanged if the analysis was restricted to women who had completed questionnaires at only one, or at every, time point during A3–A7.

### Psychosocial outcomes of alternative management policies

This is the first randomised controlled trial comparing psychosocial outcomes in women with low-grade abnormal cervical cytology attending colposcopy and managed by different approaches. The lack of any differences between the trial arms in significant depression or significant anxiety leads us to conclude that the short- and long-term psychosocial effects of immediate LLETZ and biopsy and selective recall do not differ. This conclusion is further reinforced by our observation of no difference between arms in the scores of women on the POSM, an instrument designed specifically to measure psychosocial outcomes associated with low-grade abnormal cervical cytology results and their management (Gray *et al*, 2005).

The only other study to investigate the psychosocial impact of alternative management strategies included 272 women with a colposcopic impression of high-grade disease (Orbell *et al*, 2004; Balasubramani *et al*, 2007). That study found that, 7 days after colposcopy, anxiety was significantly higher and relief significantly lower, among women undergoing biopsy and recall than among women who had had LLETZ at the colposcopy visit. Although not randomised, in an attempt to minimise bias, the authors matched women undergoing biopsy and recall to those who had LLETZ on severity of abnormality, age and deprivation of area of residence. However, our observation that prevalence of significant depression and anxiety pre-colposcopy differed between the trial arms serves to illustrate how intervention groups can differ in important ways even using a study design which strongly protects against bias, such as a randomised controlled trial. Fortunately, because we had measured depression and anxiety pre-colposcopy, we were able to adjust our analysis appropriately. The contrasting findings of our study and Balasubramani *et al* (2007) might also be a function of differences in the psychosocial instruments used and, importantly, the timing of assessment. Balasubramani *et al* (2007) dispatched their questionnaire within 7 days of colposcopy. At this point, it is likely that most women being managed by biopsy and recall would not have received their biopsy results and so would not have had any treatment: women in the biopsy group were, thus, in the midst of investigation and treatment, whereas those who had had LLETZ at the colposcopy visit had completed treatment. In contrast, we designed our short-term assessment so it would take place after treatment was completed in both arms (i.e., 6 weeks after the last intervention).

### Temporal trajectory of depression and anxiety

This study reveals the temporal trajectory of depression in women with low-grade abnormal cervical cytology attending colposcopy. The point prevalence of significant depression (HADS depression score ⩾8) rose from 6–7% at recruitment and pre-colposcopy to 10–11% at 24 and 30 months. This difference is statistically significant on a *post hoc* test. We informed GPs of those women who scored ⩾8 at any time point; hence, over time, some women (we do not know how many because of doctor–patient confidentiality) may have been treated for depression, which may have reduced the underlying prevalence. In light of this, the observed rise is intriguing and might suggest that the extended follow-up after colposcopy and treatment (mainly by 6-monthly cervical cytology tests until a woman has three consecutive normal tests and is returned to routine 3- or 5-yearly recall) is associated with depression for a small proportion of women. Longitudinal studies of women on long-term follow-up would be needed to further unravel this issue.

The long-term frequency of significant depression in our study was lower than in general population series in Denmark (604 women aged 30–75 years; 12% scored ⩾8), the United Kingdom (978 women aged 18–91 years; 13% scored ⩾8), and the Netherlands (2048 women aged 18–65 years; 22% scored ⩾8; Groenvold *et al*, 1999; Crawford *et al*, 2001; Andrea *et al*, 2004). In addition, in a study of 100 women undergoing colposcopy, there was little change in average depression scores between the initial visit and at 6 months and 2 years later (Hellsten *et al*, 2008). These observations suggest that receipt of an abnormal cervical cytology result, attendance for colposcopy and subsequent management does not have a strong influence on prevalence of clinically significant depression, either because these events do not themselves impact on depression, or because the information women receive (or source themselves) effectively counters adverse psychosocial effects.

The temporal pattern of anxiety was different to that for depression. Almost one-quarter of women had significant anxiety (HADS anxiety score ⩾11) at trial recruitment, shortly after receipt of the low-grade result, falling significantly to 14% at the colposcopy appointment around 4–12 weeks later, and to 8% 6 weeks after the last procedure. As anxiety is often triggered by uncertainty and anticipation of unknown adverse outcomes (Craig *et al*, 2000), this pattern is what one might expect *a priori* as the uncertainty associated with receipt of the cytology result ‘resolves’ with investigation and treatment. Two further conclusions follow from our data. First, our finding of no significant difference in anxiety 6 weeks post procedure between the management arms suggests that, for most women, undergoing colposcopic investigation and, perhaps also, treatment – irrespective of the procedure(s) received – alleviates, to some extent, the raised anxiety induced by receipt of the cytology result. Second, there is a significant rise in the anxiety levels after colposcopy and treatment, from 8% at 6 weeks post procedure to 14% at 12 months, with prevalence remaining stable thereafter. This long-term prevalence is similar to values reported in the European studies described above (UK, 15% Denmark, 12% Netherlands, 10%) (Groenvold *et al*, 1999; Crawford *et al*, 2001; Andrea *et al*, 2004). These two observations suggest that the apparent ‘resolution’ in anxiety after colposcopy is temporary, and an artefact of having undergone investigation and treatment, but that in the long-term prevalence returns to background levels.

### Short-term psychological distress following colposcopy and treatment compared with other interventions

Although the IES has been used fairly frequently to assess distress among cancer survivors (see, e.g., Kelly *et al*, 1995; Chen *et al*, 2005; Shim *et al*, 2006), it has rarely been applied in the context of screening or investigations for suspected cancer. The average distress score and the percentage with moderate or severe distress at 6 weeks post procedure in our study (mean=8.0, median=3.0; score ⩾26=8%) were higher than those measured at 1 week post endoscopy in a group of 192 individuals with Barrett's oesophagus (mean ∼3.5; ⩾26=6% Kruijshaar *et al*, 2006). Our participants also had higher distress scores than 236 individuals who had undergone lung cancer screening 6 months previously and had considered themselves, pre-screening, to have low cancer risk (mean=4.3, median=1.0; Bunge *et al*, 2008). Studies are difficult to compare because of the differences in participants’ gender and age and the timing of assessment of distress. However, it is possible to conclude that colposcopy and related interventions (irrespective of whether these are punch biopsies or loop excision) seem to provoke significant distress for a notable proportion of women, an important consideration to bear in mind when assessing the costs and benefits of cervical screening.

### Conclusions

See-and-treat has become increasingly common in the management of women with low-grade cytology in the United Kingdom (Kitchener *et al*, 1995). We have previously shown that immediate LLETZ and punch biopsies with selective recall do not differ in their ability to detect CIN2 or worse over 3 years (TOMBOLA Group, 2009a), or in their cost effectiveness (TOMBOLA Group, 2009c). See-and-treat results in more women undergoing unnecessary treatment and experiencing physical aftereffects, such as pain and bleeding (TOMBOLA Group, 2009b, 2009d). Others have found that LLETZ is associated with subsequent adverse reproductive outcomes (Noehr *et al*, 2009). This study shows that there is no difference in long- or short-term psychosocial sequelae of the two approaches. Debate as to which strategy offers the best balance between benefits and harms in women with low-grade abnormal cytology referred for colposcopy is likely to continue.

## Figures and Tables

**Table 1:** Baseline characteristics of women included in analysis, by trial arm; numbers and percentages

	**Immediate LLETZ**		**Biopsy and recall**	
	***n***	**%**	***n***	**%**
Total	487	100.0	502	100.0
				
*Clinical, socio-demographic and lifestyle factors*				
* Age (years)*				
20–29	207	42.5	216	43.0
30–39	134	27.5	135	26.9
40–49	106	21.8	105	20.9
50–59	40	8.2	46	9.2
* Recruitment cytology test*				
Mild dyskaryosis	117	24.0	121	24.1
Borderline nuclear abnormalities	370	76.0	381	75.9
* Trial centre*				
A	177	36.3	189	37.7
B	119	24.4	121	24.1
C	191	39.2	192	38.3
*Human papillomavirus status^a^*				
Not high risk	257	52.8	255	50.8
High risk	174	35.7	189	37.7
Not known^b^	56	11.5	58	11.6
*Deprivation category^c^*				
1 (least deprived)	57	11.7	70	13.9
2	77	15.8	110	21.9
3	101	20.7	69	13.8
4	147	30.2	127	25.3
5 (most deprived)	105	21.6	126	25.1
* Post secondary school education/training*				
None	124	25.7	122	24.4
Through work with formal qualifications	91	18.8	101	20.2
Qualifications other than degree from college/university	137	28.4	142	28.5
University/college degree	131	27.1	134	26.9
Not stated	4	—	3	—
* Employment status*				
Full-time paid employment	269	55.5	243	48.6
Part-time paid employment	105	21.7	123	24.6
Student	38	7.8	58	11.6
Not in paid employment	73	15.1	76	15.2
Not stated	2	—	2	—
* Marital status*				
Married/living as married	258	53.5	267	53.7
Divorced/separated/widowed	70	14.5	60	12.1
Single	154	32.0	170	34.2
Not stated	5	—	5	—
* Ethnicity*				
White	470	97.3	478	95.8
Other^d^	13	2.7	21	4.2
Not stated	4	—	3	—
* Parity*				
Never been pregnant	169	35.1	163	32.7
Have been pregnant, but no children	48	10.0	60	12.0
Have children	264	54.9	275	55.2
Not stated	6	—	4	—
* Smoking status*				
Never smoked	240	49.7	230	46.2
Former smoker	71	14.7	91	18.3
Current smoker	172	35.6	177	35.5
Not stated	4	—	4	—
* Physical activity*				
<1 Time/week	185	38.4	189	38.3
1–3 Times/week	114	23.7	111	22.5
>3 Times/week	183	38.0	193	39.1
Not stated	5	—	9	—
				
*Health locus of control* e				
* Chance*				
Lowest tertile (⩽16)	137	31.7	168	35.7
Middle tertile (17–21)	153	35.4	160	34.0
Highest tertile (⩾22)	142	32.9	142	30.2
Not completed^f^	55	—	32	—
* Internal*				
Lowest tertile (⩽25)	191	42.7	178	37.3
Middle tertile (26–28)	124	27.7	157	32.9
Highest tertile (⩾29)	132	29.5	142	29.8
Not completed^f^	40	—	25	—
* Powerful others*				
Lowest tertile (⩽14)	169	38.3	179	37.8
Middle tertile (15–19)	149	33.8	151	31.9
Highest tertile (⩾20)	123	27.9	144	30.4
Not completed^f^	46	—	28	—
				
*Personality* g				
* Neuroticism*				
Lowest tertile (−6 to −2)	198	43.4	189	39.9
Middle tertile (0–2)	154	33.8	167	35.2
Highest tertile (4–6)	104	22.8	118	24.9
Not completed^f^	31	—	28	—
* Extraversion*				
Lowest tertile (−6 to 2)	232	52.4	230	49.2
Middle tertile (4)	104	23.5	117	25.0
Highest tertile (6)	107	24.2	121	25.9
Not completed^f^	44	—	34	—

a Based on PCR analysis with GP5+/6+ consensus primers, followed by enzyme immunoassay for detection of 14 ‘high-risk’ human papillomavirus types.

b Includes women whose samples were inadequate for analysis, and women who did not have human papillomavirus test.

c Carstairs deprivation measure based on population quintiles assigned from address of residence at trial recruitment.

d Other ethnic group includes Black-Caribbean (*n*=13), Chinese (*n*=5), Indian (*n*=3), Black-British (*n*=2), Mixed race (*n*=2), Mixed White and Indian (*n*=2), Pakistani (*n*=2) and one each of Black-African, Japanese and Sri Lankan.

e Assessed by multi-dimensional health locus of control scale, which measures three dimensions of health locus of control (chance, internal and powerful others; Wallston *et al*, 1978).

f Includes women who either did not complete or only partially completed the questionnaire.

g Assessed by Eysenck's short questionnaire for the measurement of two dimensions of personality: neuroticism–stability and extraversion–introversion (Eysenck, 1958).

**Table 2:** Distress, avoidance and intrusion at 6 weeks post procedure (A3): prevalence, *z*-scores, odds ratios, 95% confidence intervals and *P*-values

	**Distress** a	**Avoidance** b	**Intrusion** c
*Prevalence*			
Immediate LLETZ	31.3%	6.0%	2.6%
Biopsy and recall	31.5%	7.3%	1.8%
*z*-score, *P*-value	−0.07, 0.947	−0.70, 0.486	0.77, 0.442
			
*Odds ratios, immediate LLETZ vs biopsy and recall*			
Adjusted OR, 95% CI^d^	0.97 (0.70–1.34)	0.79 (0.44–1.43)	1.48 (0.53–4.16)
Multivariate OR, 95% CI^e^	0.97 (0.68–1.37)	0.81 (0.40–1.63)	1.51 (0.48–4.77)
*P*-value^f^	0.848	0.557	0.478

Abbreviations: CI=confidence interval; EPQ=Eysenck personality questionnaire; HADS=hospital anxiety and depression scale; HPV=human papillomavirus; IES=impact of event scale; MHLCS=multi-dimensional health locus of control scale; OR=odds ratio.

a Percentage scoring ⩾9 on the IES.

b Percentage scoring ⩾20 on the IES avoidance subscale.

c Percentage scoring ⩾20 on the IES intrusion subscale.

d Adjusted for randomisation minimisation variables (age group, trial centre, high-risk HPV status and recruitment cytology status).

e Adjusted for minimisation variables, and the following: distress, HADS anxiety pre-colposcopy; avoidance, HADS anxiety pre-colposcopy, EPQ neuroticism, EPQ extraversion, MHLCS powerful others, ethnic group; intrusion, HADS anxiety pre-colposcopy, HADS depression pre-colposcopy.

f Likelihood ratio test *P*-value for randomisation arm, from multivariate model.

## APPENDIX

The TOMBOLA Group comprises

*Grant holders*

**University of Aberdeen and NHS Grampian, Aberdeen, Scotland**

Maggie Cruickshank (Principal Investigator 2009), Graeme Murray, David Parkin, Louise Smart, Eric Walker and Norman Waugh (Principal Investigator 2004–2009)

**University of Nottingham and Nottingham NHS, Nottingham, England**

Mark Avis, Claire Chilvers, Katherine Fielding, Rob Hammond, David Jenkins, Jane Johnson, Keith Neal, Ian Russell, Rashmi Seth and Dave Whynes

**University of Dundee and NHS Tayside, Dundee, Tayside**

Ian Duncan and Alistair Robertson (deceased)

**University of Ottawa, Ottawa, Canada**

Julian Little (Principal Investigator 1999–2004; currently Canada Research Chair in Human Genome Epidemiology)

**National Cancer Registry, Cork, Ireland**

Linda Sharp

**Bangor University, Bangor, Wales**

Ian Russell

**University of Hull, Hull, England**

Leslie Walker

*Staff in clinical sites and co-ordinating centres*

**Grampian:** Breda Anthony, Sarah Bell, Adrienne Bowie, Katrina Brown (deceased), Joe Brown, Kheng Chew, Claire Cochran, Seonaidh Cotton, Jeannie Dean, Kate Dunn, Jane Edwards, David Evans, Julie Fenty, Al Finlayson, Marie Gallagher, Nicola Gray, Maureen Heddle, Alison Innes, Debbie Jobson, Mandy Keillor, Jayne MacGregor, Sheona Mackenzie, Amanda Mackie, Gladys McPherson, Ike Okorocha, Morag Reilly, Joan Rodgers, Alison Thornton and Rachel Yeats

**Tayside**: Lindyanne Alexander, Lindsey Buchanan, Susan Henderson, Tine Iterbeke, Susanneke Lucas, Gillian Manderson, Sheila Nicol, Gael Reid, Carol Robinson and Trish Sandilands

**Nottingham**: Marg Adrian, Ahmed Al-Sahab, Elaine Bentley, Hazel Brook, Claire Bushby, Rita Cannon, Brenda Cooper, Ruth Dowell, Mark Dunderdale, Dr Gabrawi, Li Guo, Lisa Heideman, Steve Jones, Salli Lawson, Zoë Philips, Christopher Platt, Shakuntala Prabhakaran, John Rippin, Rose Thompson, Elizabeth Williams and Claire Woolley

*Statistical analysis* Seonaidh Cotton, Kirsten Harrild, John Norrie and Linda Sharp

*External Trial Steering Committee*

Nicholas Day (chair, 1999–2004), Theresa Marteau (chair, 2004), Mahesh Parmar, Julietta Patnick and Ciaran Woodman.

*External Data Monitoring and Ethics Committee*

Doug Altman (chair), Sue Moss and Michael Wells

**Writing and analysis group for this paper**

Linda Sharp, Seonaidh Cotton, Nicola Gray, Mark Avis, Ian Russell, Leslie Walker, Norman Waugh, David Whynes, Claire Woolley, Alison Thornton, Louise Smart, Maggie Cruickshank and Julian Little

**Disclosure of interests for writing and analysis group**

JL has received fees from GlaxoSmithKline as a member of an Independent Data and Safety Monitoring Committee for a trial of the efficacy of vaccination against HSV. None of the other members of the writing and analysis group have any relevant interests to declare.

**Funding and role of funders**

This trial was funded by the Medical Research Council and NHS in Scotland and England. The funders had no role in study design, collection, analysis and interpretation of the data, in writing this article or in the decision to submit for publication.

**Trial Registration**

ISRCTN 34841617 (http://www.isrctn.org/).
